# Osteoclasts directly influence castration-resistant prostate cancer cells

**DOI:** 10.1007/s10585-022-10179-2

**Published:** 2022-08-16

**Authors:** Junchi Huang, Eva Freyhult, Robert Buckland, Andreas Josefsson, Jan-Erik Damber, Karin Welén

**Affiliations:** 1 Department of Urology, Institute of Clinical Sciences, Sahlgrenska Center for Cancer Research, Sahlgrenska Academy, University of Gothenburg, Gothenburg, Sweden; 2 Department of Cell and Molecular Biology, Science for Life Laboratory, National Bioinformatics Infrastructure Sweden, Uppsala University, 75124 Uppsala, Sweden; 3 Department of Surgical and Perioperative Sciences, Umeå University, Urology & Andrology, Umeå, Sweden; 4 Wallenberg Center for Molecular Medicine (WCMM), Umeå University, Umeå, Sweden

**Keywords:** Castration-resistant prostate cancer, Osteoclasts, RNA sequencing, DNA repair, Apoptosis

## Abstract

Metastasis to bone is the leading cause of death from prostate cancer. Interaction between tumor cells and bone cells can promote progression and influence tumor phenotype. It is known that prostate cancer cells support osteoclast differentiation, and degradation of bone matrix by osteoclasts releases growth factors stimulating tumor cell proliferation and invasion. In the present study osteolytic (PC-3) and osteoblastic (LNCaP-19) castration-resistant prostate cancer (CRPC) cells were co-cultured with mature osteoclasts or their precursor cells (RAW 264.7) to characterize direct effects of mature osteoclasts on CRPC cells. Osteoclasts increased proliferation and decrease apoptosis of CRPC cells as assessed with flow cytometry. RNA sequencing revealed that osteolytic CRPC cells were more responsive to osteoclast stimulation regarding gene expression, but the overall induced expression patterns were similar between the prostate cancer cell lines. Genes related to DNA repair were upregulated by osteoclasts, while genes related to endoplasmic reticulum stress-induced apoptosis and cholesterol synthesis were downregulated. The results of this study shows that osteoclasts directly influence CRPC cells, increasing proliferation, decreasing apoptosis, and affecting gene expression pathways that can affect sensitivity to DNA damage and endoplasmic reticulum function. This suggests targeting of osteoclasts to be a possible way to affect efficacy of other drugs by combination regimens in treating prostate cancer metastases.

**Supplementary Information:**

The online version contains supplementary material available at 10.1007/s10585-022-10179-2.

## Introduction

Prostate cancer metastasis to bone is the major cause for morbidity and mortality of prostate cancer [1]. Prostate cancer cells form metastatic lesions characterized by increased bone formation [2, 3]. This contrasts to other cancer forms where skeletal metastasis is common, such as lung and breast cancer, where osteolytic lesions dominate [4]. However, in prostate cancer the resulting osteogenic phenotype, where bone marrow is suppressed by newly formed osteoid, still includes osteolytic areas, and ongoing osteolysis is a prerequisite for the increased osteogenic process [2, 5].

Osteoclasts and osteoblasts regulate their reciprocal action, leading to the balance of continuous formation and degradation of bone maintaining the bone structure intact. Osteoblasts secrete RANKL, which binds to its receptor on osteoclast precursors initiating the differentiation into mature osteoclasts, a process regulated by M-CSF as a rate limiting factor. Multinucleated osteoclasts sit on the bone surface releasing release hydrogen ions, collagenase, cathepsin K and other hydrolytic enzymes. The acidic environment increases solubility of bone mineral and the enzymes digest and degrade collagen and other organic components of the decalcified bone matrix which releases growth factors previously bound to the bone matrix [6].

Metastatic prostate cancer is treated with androgen deprivation therapy (ADT), most often gonadotropin-releasing hormone (GnRH) agonists, to block testosterone synthesis and inhibit activation of the androgen receptor (AR), a mechanism critical for survival and proliferation of prostate cancer cells. In the majority of patients, this decreases tumor burden, and prolongs time to progression, although eventually castration-resistant prostate cancer (CRPC) relapses. However, ADT also causes a decrease in bone mineral density since androgens [7], especially by their conversion into estrogens, are important for the maintenance of bone. Estrogen inhibits bone resorption by osteoclasts by shifting the balance of osteoprotegerin (OPG)/ RANKL secretion from osteoblasts as well as limiting the number of tumor necrosis factor-producing T-cells [8–12]. Degradation of bone by osteoclasts in osteoporosis leads to release of several growth factors. This promotes proliferation of resident prostate cancer cells, in a pattern described as a vicious cycle, since the tumor cells in turn, via secretion of parathyroid hormone related protein, promote osteoblasts to secrete RANKL increasing osteoclastic activity [13, 14].

Inhibition of osteoclast differentiation and activation, by blocking of RANKL signaling or RANK expression has been shown to decrease osteolytic prostate cancer growth in bone in experimental in vivo settings [15–17]. In humans, therapy inducing osteoclast apoptosis [18] and RANKL-inhibitors aiming to inhibit osteoclast differentiation and activation is frequently used among prostate cancer patients in order to increase bone strength and decrease bone fractures. Both are used with approximately the same efficacy [19]. Denosumab, an inhibitory RANKL antibody, was demonstrated to increase bone metastasis-free survival [20] and to delay skeletal related events in patients with CRPC [21]. However, no survival benefit for prostate cancer patients could be determined [19, 20]. Another possible treatment option for prostate cancer patients, besides different forms of androgen inhibition, is the PARP-inhibitors targeting cells with impaired DNA repair mechanism [22].

The indirect stimulatory role of osteoclasts on metastatic growth by degradation of bone has been acknowledged in prostate cancer, despite the osteogenic phenotype of the metastases. Also, several studies demonstrate the promoting effect of prostate cancer cells on osteoclastogenesis [23, 24]. Osteoblasts influence CRPC cells’ expression of osteogenic genes [25]. Furthermore, osteogenic CRPC cells responded to osteoblast stimulation with increased proliferation and osteogenic properties [25], as well as expression of genes encoding steroidogenic enzymes [26]. It has also been reported that bone-derived stroma cells can influence AR signaling in CRPC cells [27]. A recent study shows that Wnt-activation of RAW 264.7 derived osteoclasts promoted migration and invasion of breast cancer cells, an effect that could be inhibited by activation of the Wnt pathway in the osteoclasts [28]. In addition, we have recently reported that osteoclasts can influence steroidogenesis in prostate cancer cells [29]. However, the influence of osteoclasts on prostate cancer cell proliferation and gene expression has previously not been extensively investigated.

In the present study, we analyze the effects of osteoclasts on proliferation and survival of CRPC cells and use RNA sequencing to illustrate the gene expression changes in CRPC cells induced by osteoclasts, together with a focused study on genes related to bone metabolism. This will define any direct influence of osteoclasts on CPRC progression as bone metastases.

## Material and methods

### Cell culture

The osteogenic castration-resistant cell line LNCaP-19 is developed from LNCaP and has been characterized previously [25, 30, 31]. Osteolytic CRPC PC-3 cells were obtained from the European Collection of Cell Cultures ECCC (Wiltshire, UK). LNCaP-19 and PC-3 cells were maintained in RPMI 1640 medium supplemented with glucose, sodium pyruvate medium and 10% charcoal–dextran stripped serum ((CSS), Invitrogen) or 10% fetal bovine serum ((FBS), Invitrogen). Murine RAW 264.7 cells, osteoclast precursors, were obtained from ATCC (ATCC TIB-71) (Rockville MD) and maintained in DMEM (ATCC® 30–2002™) with 10% FBS. All cultures were supplemented with 1% penicillin/streptomycin and confirmed mycoplasma free.

### Formation of osteoclasts

RAW 264.7 macrophages were transformed into osteoclasts by the stimulation of soluble RANKL (EMD Millipore GF091). The RAW 264.7 cells were seeded with 5 × 10^3^ cells/cm^2^ concentration with 10 ng/ml of sRANKL in DMEM (ATCC 30–2002) with 10% FBS in six well plates. Medium (including sRANKL) was changed every 72 h for 6 days. At day 7, cells were washed with PBS and fixed with 4% paraformaldehyde for 15 min. Mature osteoclasts were defined by positivity for tartrate-resistant acid phosphatase (TRAP) positivity using the Acid Phosphatase, Leukocyte (TRAP) Kit (Sigma-Aldrich 387A-1KT).

### Co-culture of osteoclasts and prostate cancer cells

Co-culture with osteoclasts and LNCaP-19 or PC-3 was performed using the Transwell permeable support (24 mm insert, 0.4 µm pore size, polyester membrane) in 6 well plates (Corning 3450, Kennebunk, ME, USA). Pre-incubation of Transwell inserts with the culture medium for LNCaP or PC-3 cells, as specified above, was performed for 24 h before seeding of 3 × 10^5^ cells/insert of PC-3 or LNCaP-19 cells on the membrane. After 36 h, the inserts with PC cells were moved into plates with mature osteoclast (after the formation procedure described above) in the lower compartments. RAW 264.7 cells were seeded 5 × 10^3^ cells/cm^2^ in control wells at the start of osteoclast maturation. Both osteoclasts and RAW 264.7 cells were washed with PBS, and LNCaP-19 or PC-3 culture medium (without sRANKL) was added prior to start of the co-culture. Co-culturing was performed for 48 h after which cells were harvested for later analysis. All culture experiments were repeated at least three times.

### UV-treatment

UV-C irradiation is able to induce highly efficiently DNA damage [32]. LNCaP-19 or PC-3 cells were cultured in transwells for 36 h as specified in co-culture section. The culture medium was removed before the UV-C treatment, cells were irradiated for 1 min, and then the medium was added immediately after the treatment. The transwells with UV treated PC cells were moved into plates with mature osteoclast or untreated RAW 264.7 cells as controls in the lower compartments. After 48 h of co-culturing, PC cells were harvested for DNA damage analysis with Western blotting using phosphorylated histone protein γ-H2AX as a marker for DNA strand breaks.

### Flow cytometry

For cell apoptosis assay, after 48 h co-culturing, the cells were collected and washed in ice-cold PBS. PE Annexin V Apoptosis Detection kit (BD Pharmingen™, USA) was used to assess cell apoptosis according to the manufacturer’s instructions. 7-AAD was used to stain dead cells. The cells were incubated with 5 µl of PE Annexin V and 5 µl 7-AAD for 15 min at RT (25 °C) in the dark. Unstained cells, cells stained with PE Annexin V (no 7-AAD) and cells stained with 7-AAD (no PE Annexin V) were used to set up compensation and quadrants.

For the cell proliferation assay, EdU Staining Proliferation Kit (iFluor 488) (Abcam, UK) was used to analyze the proliferation of cells after 48 h co-culturing. EdU solution was added and incubated together with the cells for 4 h under optimal growth conditions. Both cell apoptosis and cell proliferation were analyzed with a flow cytometer (BD Accuri™ C6, BD Biosciences).

### RNA isolation and RNA sequencing

Total RNA was isolated using RNeasy Mini Plus kit (Qiagen, USA) according to the manufacturer’s instructions. RNA concentration was measured on a NanoDrop (Thermo Fisher, USA). RNA samples from LNCaP-19 cells and PC-3 cells co-cultured with mature osteoclasts or control cells (RAW 264.7 cells) were quantified and checked for contamination using (Dropsense96, Trinean). After normalization for equal input (500 ng in 5 ul), samples were spiked with ERCC RNA Spike-In Control Mix (ThermoFisher). Libraries were prepared using QuantSeq 3′ mRNA-Seq (FWD) Library Prep Kit for Illumina (Lexogen) including individual indexing for multiplex sequencing. Finalized libraries were quality checked with capillary electrophoresis (Fragment Analyzer) and quantified with Picogreen (ThermoFisher). The libraries were normalized and pooled before NextSeq500 sequencing (Illumina).

### Protein preparation and Western blotting

The cells were lysed with RIPA Lysis and Extraction Buffer (Invitrogen™, USA) supplemented with PhosSTOP™ phosphatase inhibitor and cOmplete™ protease inhibitor cocktail (Roche Diagnosis; DE). The protein concentration was determined using a Coomassie Plus (Bradford) Assay Kit (Pierce™, USA) according to the manufacturer’s instructions. Protein lysates were separated by NuPAGE™ 4–12%, Bis–Tris gel and then transferred to PVDF membranes (Invitrogen™, USA). Membranes were blocked in Pierce™ Clear Milk Blocking Buffer (Pierce Biotechnology; USA) and incubated with primary antibodies (Supplementary X) at 4 °C overnight. Then the membranes were washed with PBS-T and incubated with secondary antibodies for 1 h at RT (Antibodies are specified in Supplementary Table 1). Amersham™ ECL Select™ western blotting detection reagent (GE Healthcare; UK) was used for visualizing and detection following manufacturer’s instruction.

### Data handling and statistics

The pipeline Human (GRCh38) Lexogen QuantSeq 2.6.1 was used for the sequence data analysis. After mapping the reads against the ENSEMBL reference genome, we merged the reads count files using a custom bash script. ENSEMBL identifiers were translated into HGNC gene names using a custom R script that retrieved the corresponding gene names from the BioMart database. Differential gene expression was performed using the R-package edgeR [33]. Multiple testing correction is performed using Benjamini–Hochberg false discovery rate (FDR) method. A gene is considered differentially expressed if FDR < 5% and the log-transformed fold change (log2(FC)) > 1 (up-regulated) or log2(FC) <  − 1 (down-regulated). All non-protein coding RNAs were removed prior to analysis. Lists of significantly affected genes were assessed for gene ontology (GO) enrichment using PANTHER Overrepresentation Test (released 2020-07-28) and the PANTHER Enrichment test annotated with GO Ontology database https://doi.org/10.5281/zonodo.4081749 (released 2020-10-09) [34]. The annotation Data sets used was GO biological process complete and Reactome Pathways. The analysis was performed using the Fisher’s Exact test with Bonferroni correction for multiple testing. Only GO terms with a Bonferroni adjusted *P*-value < 0.05 were considered. QIAGEN IPA (QIAGEN Inc., https://digitalinsights.qiagen.com/IPA) software was used for further analysis of upstream regulators and for illustration of affected pathways in the significant genes. Differences in gene expression in the osteogenesis PCR array were statistically evaluated using Student’s t-test. These calculations were performed in IBM SPSS Statistics 26. Flow cytometry results were handled in the BD Accuri TM C6 analysis software and statistically evaluated with unpaired t-test with Welch’s correction using the GraphPad Prism 9.0.0.

## Results

### Osteoclasts diminish apoptosis and promote proliferation in CRPC cells

**Fig. 1:**
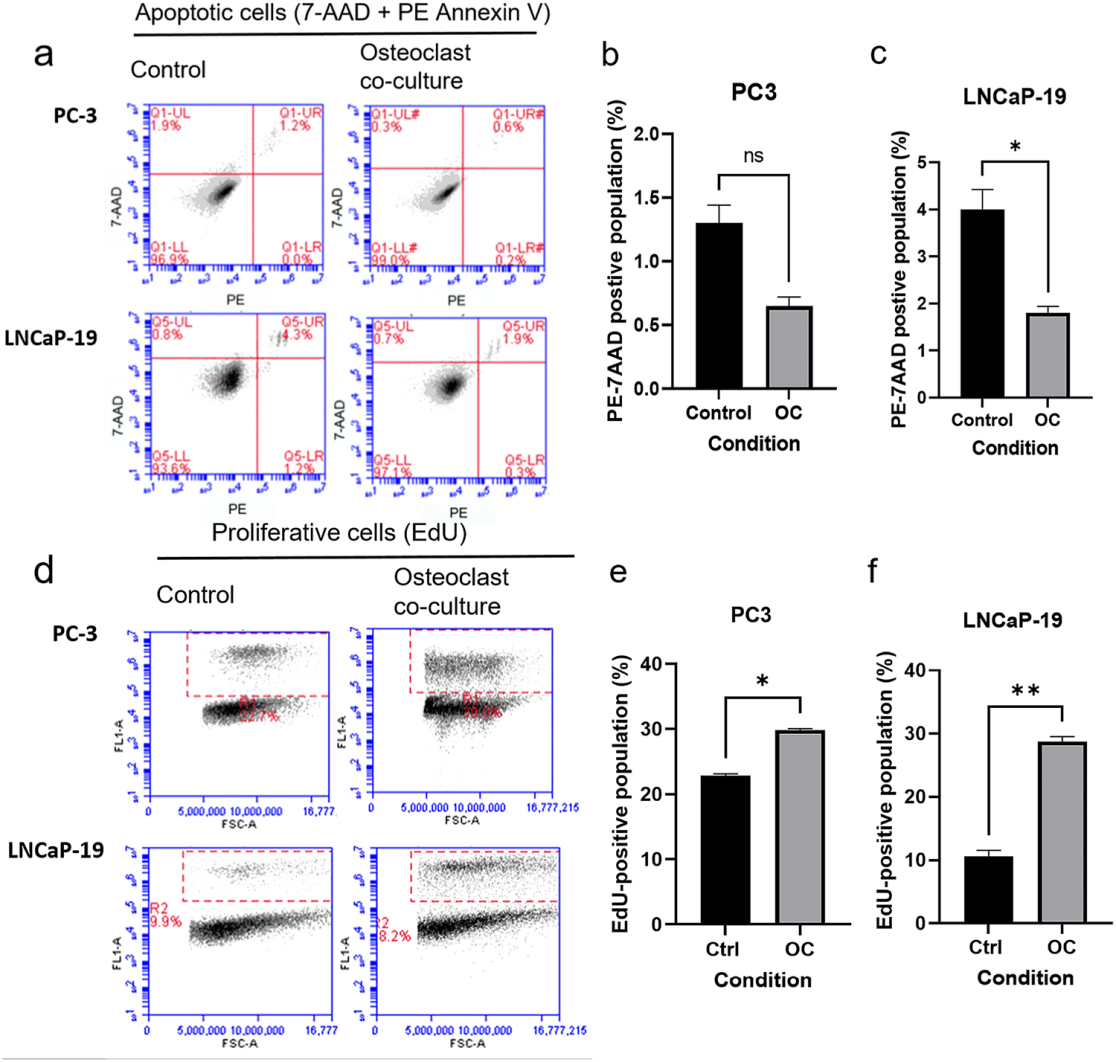
Effects of osteoclasts on prostate cancer cell apoptosis and proliferation **a** Representative images of the flow cytometry of PC-3 and LNCaP-19 cells labelled with 7AAD and PE Annexin V after 48 h co-culture with mature osteoclasts or control cells (unstimulated RAW 264.7 cells). **b**, **c** Effects on osteoclasts on apoptosis (7AAD^+^ and Annexin V^+^) in PC-3 (**b**) and LNCaP-19 (**c**). Bars represent mean ± SEM. **d** Representative images of the flow cytometry of PC-3 and LNCaP-19 cells labelled with EdU after 48 h co-culture with osteoclasts or control cells. **e**, **f** Effects on osteoclasts on proliferation (EdU^+^) in PC-3 (**e**) and LNCaP-19 (**f**)

Osteolysis is known to stimulate proliferation of metastatic cells by release of growth factors bound to bone tissue. To investigate any direct effect of mature osteoclasts on PC cell proliferation or apoptosis, a flow cytometry assay was used on PC cells after co-culturing with mature osteoblasts or control RAW cells. The apoptosis assay using Annexin V and 7-AAD showed a decrease of end stage apoptotic cells in LNCaP-19 co-cultured with osteoclasts compared to the control condition, while in PC-3 cells, the decrease in apoptosis was not statistically significant (Fig. 1a–c). Furthermore, mature osteoclasts promoted proliferation in both LNCaP-19 and PC-3 cells using an EdU incorporation assay (Fig. 1d–f).

### The majority of osteoclast-induced expression changes are upregulation

To further investigate the influence of osteoclasts on CRPC cells, analysis of the gene expression changes induced by the co-culture was performed with RNA-sequencing. A principal component analysis of the RNA sequencing data showed that the two cell lines were well separated and samples from co-cultures with mature osteoclasts and the macrophage control cells were separated within the individual clusters for the cell lines (Fig. S1).

The sequencing generated read counts across 56,324 genes. After filtering out genes with low counts (< 2 cpm in the majority of samples) that was reduced to 13,511. Of these, 6226 (46%) were significantly affected by osteoclast stimulation (FDR < 0.05). Although non-protein coding types of RNA have been shown to influence osteoclasts and bone metabolism [35, 36], the result in the present study is focused on the protein coding genes.

In total, the expression of 3566 protein coding genes was significantly affected by co-culture with mature osteoclasts compared to precursor cells (FDR < 0.05, −1 > log2(FC) > 1). Gene expression in PC-3 was generally more responsive to osteoclast stimulation with 2756 affected genes, compared to 1479 in LNCaP-19. Of the 3566 genes, approximately 20% (669 genes) responded with a change in the same direction in both cell lines. In general, both cell lines responded with more increased gene expression than decreased (in total 2100 upregulated and 1466 downregulated) (Fig. S1).

### Gene ontology and pathway analysis

GO overrepresentation analyses revealed that osteoclast stimulation of LNCaP-19 and PC-3 cells affect largely the same groups of genes in both cell lines. GO terms that appear as overrepresented for both PC-3 and LNCaP-19 were *cell cycle, chromosome organization, DNA repair, DNA replication, tRNA processing/metabolic process,* and *macromolecule methylation/modification*. Underrepresented in both was the *G-protein-coupled receptor signaling pathway* (Table 1a, b). Adding the fold change of the altered genes into the analysis, the PANTHER enrichment test revealed differences between the cell lines’ response to osteoclasts. This test compares the average fold change of genes within an identified GO term with the average fold change of the whole data set. PC-3, due to its larger amount of affected genes, presented with a broader range of enriched GO terms; the only positively enriched GO terms were associated with RNA modification, while the list of negatively enriched GO terms was topped by *regulation of apoptosis*, *regulation of cell differentiation*, and *regulation of cell population proliferation*. In LNCaP-19, only four GO terms were identified with the PANTHER enrichment test. Positively enriched were *double-strand break repair *via* homologous recombination* and *DNA-templated DNA replication, while response to endoplasmic reticulum stress* and *regulation of epithelial cell differentiation* displayed a more downregulated profile compared to the whole data set (Table 2a, b).

**Table 1:** Overepresented gene ontology terms based on gene expression in PC-3 (A) and LNCaP-19 (B) cells influenced by osteoclasts

	Number of genes	Expected number of genes	Fold Enrichment	*P* value
A. GO biological process complete				
*Overrepresented in PC-3*				
Chromosome organization	198	122.57	1.62	2.04E−05
Cell cycle	239	159.26	1.50	1.17E−04
Cellular protein modification process	463	354.57	1.31	2.42E−04
Negative regulation of transcription by RNA polymerase II	195	126.08	1.55	4.84E−04
Organonitrogen compound biosynthetic process	251	173.19	1.45	7.58E−04
Cellular macromolecule biosynthetic process	159	99.41	1.60	1.51E−03
Intracellular transport	248	174.75	1.42	3.87E−03
Cellular component biogenesis	439	342.21	1.28	4.16E−03
Positive regulation of cellular metabolic process	535	433.16	1.24	1.07E−02
DNA repair	109	63.76	1.71	1.23E−02
DNA replication	60	28.50	2.11	1.53E−02
tRNA processing	42	16.92	2.48	1.64E−02
Macromolecule methylation	69	35.78	1.93	3.27E−02
Positive regulation of nitrogen compound metabolic process	503	408.83	1.23	3.71E−02
Carbohydrate derivative biosynthetic process	124	77.94	1.59	4.36E−02
*Underrepresented ni PC-3*				
G protein-coupled receptor signaling pathway	72	158.87	.45	6.23E−10
Adaptive immune response	29	84.84	.34	1.75E−07
B. GO biological process complete				
*Overrepresented in LNCaP-19*				
Cell cycle	141	85.43	1.65	4.16E−04
Chromosome organization	113	65.75	1.72	1.68E−03
Cellular amino acid metabolic process	48	19.61	2.45	1.77E−03
DNA repair	70	34.20	2.05	1.91E−03
DNA replication	40	15.29	2.62	3.24E−03
Regulation of apoptotic process	154	101.62	1.52	1.17E−02
Regulation of cellular response to stress	87	49.48	1.76	2.17E−02
Regulation of gene expression	418	337.32	1.24	2.24E−02
Negative regulation of cellular process	420	339.69	1.24	2.38E−02
tRNA metabolic process	34	13.26	2.56	3.57E−02
Regulation of RNA biosynthetic process	312	241.84	1.29	3.79E−02
Macromolecule modification	271	205.75	1.32	4.59E−02
*Underrepresented in LNCaP-19*				
G protein-coupled receptor signaling pathway	28	85.22	.33	1.13E−08

**Table 2:** Gene set enrichment and pathway analysis based on fold change of altered genes in PC-3 (A) and LNCaP-19 (B) after osteoclast co-culture

	Number of genes	Direction of enrichment	*P* value
A. GO biological process complete in PC-3			
ncRNA processing	104	+	1.92E−02
RNA modification	42	+	4.86E−02
Regulation of apoptotic process	245	**–**	7.23E−05
Regulation of cell differentiation	229	**–**	4.93E−04
Regulation of cell population proliferation	260	**–**	8.66E−04
Regulation of MAPK cascade	106	**–**	1.01E−03
Response to starvation	43	**–**	3.37E−03
Negative regulation of intracellular signal transduction	98	**–**	4.21E−03
Negative regulation of cellular protein metabolic process	149	**–**	4.31E−03
Tissue development	250	**–**	4.61E−03
Apoptotic process	151	**–**	5.07E−03
Muscle structure development	72	**–**	8.55E−03
Positive regulation of cellular protein metabolic process	227	**–**	9.86E−03
Regulation of hydrolase activity	122	**–**	9.86E−03
Response to hypoxia	56	**–**	1.24E−02
Cellular response to nutrient levels	47	**–**	1.47E−02
Cellular response to hormone stimulus	78	**–**	2.23E−02
Response to growth factor	73	**–**	3.67E−02
Reactome pathways in PC-3			
Activation of gene expression by SREBF (SREBP)	16	**–**	3.13E−02
Cellular response to starvation	27	**–**	3.90E−02
Response of EIF2AK1 (HRI) to heme deficiency	10	**–**	4.19E−02
B. GO biological process complete in LNCaP-19			
DoublE−strand break repair via homologous recombination	21	** + **	8.40E−03
DNA-templated DNA replication	29	** + **	9.70E−03
Response to endoplasmic reticulum stress	24	**–**	4.23E−02
Regulation of epithelial cell differentiation	21	**–**	4.50E−02
Reactome pathways in LNCaP-19			
DNA strand elongation	14	** + **	9.17E−03
Unfolded Protein Response (UPR)	17	**–**	1.43E−02

Using the Reactome Pathway option built into the PANTHER tool, enriched pathways were identified in the cell lines after co-culture with osteoclasts. In LNCaP-19, only two pathways were identified (Table 2b). In PC-3 however, some distinct pathways not clearly overlapping with the enriched GO terms appear. The most significantly affected pathway was *activation of gene expression by SREBF*, which regulates cholesterol biosynthesis (Table 2a). A parallel Core Analysis of fold change in significantly altered genes was carried out in QIAGEN IPA to identify affected pathways in an untargeted fashion for each cell line. As with the previous analyses, pathways related to cancer (cell cycle, DNA replication and cell death) were identified by IPA as being the most affected by co-culture with osteoclasts, especially in LNCaP-19. In PC-3, a partly different picture emerges including the SREBP2 pathway (Graphical illustration in Supplementary Fig. S3). IPA was then used to generate illustrative pathways using a gene list for genes coupled to SREBF2 (Supplementary Fig. S4). All 18 genes in the pathway were significantly altered when PC3 cells were co-cultured with osteoclasts, whereas only 9 of the 18 were significantly altered in LNCaP-19 cells (Supplementary Table 2).

### The most significantly affected genes

Studying the most significantly affected genes (based on lowest false discovery rate), it appears that the upregulated profile in PC-3 is the most specific response to osteoclast secretory products. Of the 20 most significantly upregulated genes in PC-3, 14 were not affected in LNCaP-19. In comparison, 16 of the top 20 upregulated genes in LNCaP-19 were also upregulated in PC-3. No such difference was observed among the top 20 downregulated genes, where most of the genes were affected in both cell lines. Eight genes (DDIT3, DDIT4, GDF15, GADD45B, ATF3, SAT1, BBC3, and INSIG1) appear on the top 20 lists for both cell lines, indicating a strikingly similar pattern for the most downregulated genes in response to osteoclast stimulation (Supplementary Table 3). In line with this data, a DDIT3 centered network cluster was identified in the QIAGEN IPA analysis in both cell lines limiting the analysis to genes with fold change > 3 (Supplementary Fig. S5).

In line with the GO and pathway analyses, many of the most upregulated genes, especially in LNCaP-19, were related to regulation of RNA. Five genes for heterogeneous nuclear ribonucleoproteins (HNRNPM, HNRNPP2, HNRNPD, HNRNPAB, and HNRNPDL) which complex with heterogeneous nuclear RNAs were found, together with additionally at least three genes involved in RNA splicing (SFPQ, SRSF3, U2AF2). The homogenous pattern regarding the most downregulated genes in both cell lines clearly matched the GOs and pathways related to ER-induced stress response, where BBC3, DDIT3, ATF3, ATF4, and CHAC1 all co-operate in apoptosis induced by ER-stress (Supplementary Table 3).

### Upstream regulators

The QIAGEN IPA software was used to identify possible upstream regulators for the changes induced by osteoclasts in PC-3 and LNCaP-19 (Table 3a, b). This module in IPA uses identified pathways integrated with previously reported associations in the literature to identify putative upstream regulators of the observed effects. In PC-3, the transcriptional regulators NUPR1, HNF4A, and p53 could possibly mediate the effects on apoptosis and DNA repair. In LNCaP-19, ATF4 was identified as a possible upstream inducer of the ER stress related effects, while the inhibited action of EIF2AK3 could contribute to the regulation of expression of many types of genes. Suggested as a common upstream regulator for PC-3 and LNCaP-19 was the mTOR inhibitor 2-(4-amino-1-isopropyl-1H-pyrazolo[3,4- d]pyrimidin-3-yl)-1H-indol-5-ol (TORKinib or PP242), a mammalian target of rapamycin (mTOR) inhibitor, whose action was predicted by the IPA software to be inhibited.

**Table 3:** Putative upstream regulators identified by IPA in PC-3 (A) and LNCaP-19 (B)

A. Regulators in PC-3	*P* value	Predicted activation	Function
NUPR1	1.28 × 10^30	Inhibited	Transcription regulator in cell-cycle, apoptosis, autophagy and DNA repair responses
HNF4A	1.47 × 10^19		Nuclear receptor, induces senescence in PC
2-(4-amino-1-isopropyl-1H-pyrazolo[3,4- d]pyrimidin-3-yl)-1H-indol-5-ol	3.26 × 10^16	Inhibited	mTOR inhibitor
l-asparaginase	1.11 × 10^15		Metabolizes L-asparagine, leukemia drug
TP53	5.64 × 10^15	Inhibited	Tumor suppressor
B. Putative regulators in LNCaP-19			
2-(4-amino-1-isopropyl-1H-pyrazolo[3,4- d]pyrimidin-3-yl)-1H-indol-5-ol	1,99 × 10^17	Inhibited	mTOR inhibitor
EIF2AK3	3,28 × 10^16	Inhibited	Inactivates transcription
Tosedostat	6,01 × 10^16	Inhibited	Inhibits M1 aminopeptidases. Anti-cancer drug
Nelfinavir	8,39 × 10^16	Inhibited	HIV drug with anticancer properties
ATF4	1,21 × 10^14	Inhibited	trancription factor, regulates ER-stress with DDIT3

### Downregulation of proteins in cholesterol synthesis pathway

**Fig. 2:**
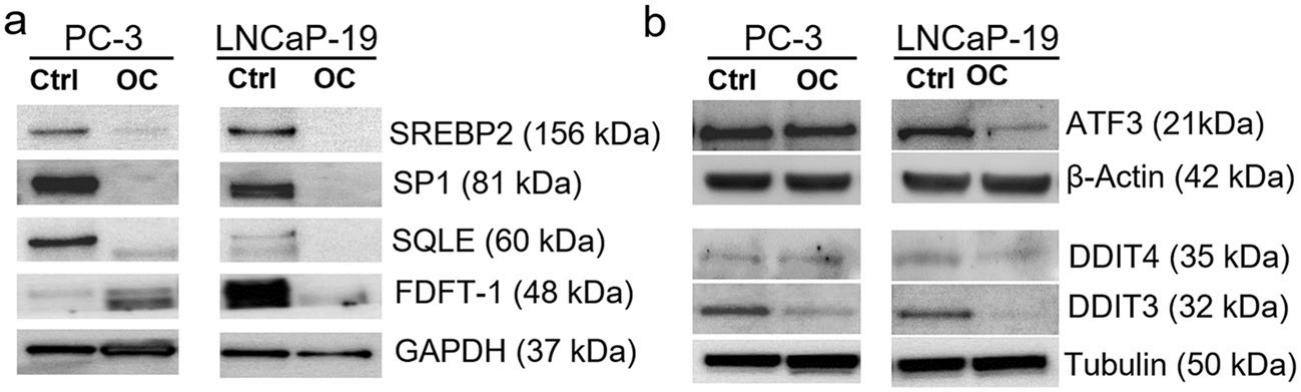
Protein analysis of selected genes **a** Western blotting of selected proteins in the SREBP2/cholesterol pathway, and **b** Western blotting of selected proteins in the ER stress induced apoptosis pathway in PC-3 and LNCaP-19 cells after co-culture with mature osteoclasts or control cells (unstimulated RAW 264.7 cells). GAPDH, β-actin and tubulin were used as loading controls

Cholesterol is an important component of cell membranes as well as the precursor for steroid synthesis. The RNA-sequencing data indicated that osteoclast simulation downregulates SREBP2 regulated genes, of which several are enzymes involved in cholesterol synthesis, such as 3-Hydroxy-3-Methylglutaryl-CoA Synthase 1 (HMGCS1), Farnesyl-Diphosphate Farnesyltransferase 1 (FDFT1), and Farnesyl Diphosphate Synthase (FDPS) [37]. To verify the effects of osteoclasts on this pathway, selected proteins were investigated by Western blotting. Among the selected genes, SRBEF2 itself and its co-transcription factor SP-1, as well as squalene epoxidase (SQLE), which is one of the rate-limiting enzymes in this pathway, were downregulated after osteoclast stimulation in both PC-3 and LNCaP-19. Conversely, FDFT1, the first specific enzyme in cholesterol biosynthesis was upregulated in PC-3 (Fig. 2a).

### Downregulation of proteins related to ER-induced stress response

To investigate whether the osteoclasts-induced downregulation of the proteins related to the ER-stress induced apoptosis was translated into changes also on the protein level, Western blotting analysis was performed. Among the selected genes, DDIT3 which induces cell cycle arrest and apoptosis in response to ER stress [38] was downregulated by co-culture with osteoclasts at the protein level in both cell lines, while the transcription factor ATF3 was downregulated only in LNCaP-19 (Fig. 2b).

### DNA damage after UV exposure of CRPC cells is affected by osteoclasts

**Fig. 3:**
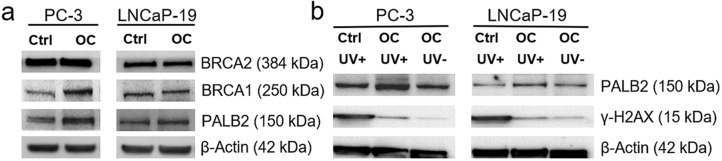
Protein analysis of DNA repair pathway **a** Western blotting of selected proteins in the DNA repair pathway in PC-3 and LNCaP-19 after co-culture with mature osteoclasts or control cells (unstimulated RAW 264.7 cells), and **b** accumulation of γ-H2AX as a marker for DNA breaks in UV-exposed PC-3 and LNCaP-19 cells after co-culture with mature osteoclasts or control cells (unstimulated RAW 264.7 cells). β-actin was used as loading control

To investigate whether the induction of expression of genes related to DNA repair could be translated into the protein level, Western blotting analysis was performed on selected genes in this GO group. Two of the analyzed proteins, BRCA1 and PALB2 was increased in PC-3, while the effect was not seen as clearly in LNCaP-19 after co-culture with osteoclasts compared to the control condition. The expression level of BRCA2 was not affected by osteoclasts on the protein level (Fig. 3a). To investigate whether this altered expression of genes related to DNA repair by osteoclasts influences the accumulation of DNA breaks, we exposed LNCaP-19 and PC-3 cells to UV-light and analyzed the amount of DNA breaks after 48 h of co-culture. In both cell lines, co-culture with osteoclasts results in less DNA breaks, reflected by the lower levels of accumulated γ-H2AX compared to cells co-cultured with precursor RAW 264.7 cells (Fig. 3b).

### Genes related to bone metabolism

To specifically study the effects of osteoclasts on genes expression related to bone metabolism and enable comparisons with the effects of osteoblasts previously described [25], an osteogenesis directed PCR panel was used. Similar to the RNA sequencing results, PC-3 was more sensitive to osteoclast simulation compared to LNCaP-19, with 12 genes statistically changed (2FC 0.05) compared to only one (TGFB2) in LNCaP-19 (Table 4). These changes indicate a pattern where osteoclasts induce PC-3 cells to express genes for secreted factors that promote osteogenesis (BMP6, VEGFA, TGFB2) and inhibit osteoclast differentiation and activation (CSF2), while decreasing expression of CSF3, which stimulates osteoclastogenesis and induces apoptosis in osteoblasts [39].

**Table 4:** Differential gene expression in PC-3 and LNCaP-19 co-cultured with osteoclasts

Gene ID	Fold change		Gene name
	PC-3	L-19	
ALPL	3.25	1.97	Alkaline phostphatase
ARSE	0.16	ND	Arylsulphatase E
BMP1	1.82**	=	Bone morphogenetic protein 1
BMP2	=	0.43	Bone morphogenetic protein 2
BMP4	0.64*	0.46	Bone morphogenetic protein 4
BMP6	4.14**	=	Bone morphogenetic protein 6
BMP7	1.87**	=	Bone morphogenetic protein 7
CDH11	0.43**	=	Cadherin 11 / OB-cadherin
COL2A1	0.27*	ND	Collagen, type II, alpha 1
COL17A1	0.49**	2.43	Collagen, type XVII, alpha 1
COL18A1	2.08*	=	Collagen, type XVIII, alpha 1
CSF2	4.01**	ND	Colony stimulating factor 2
CSF3	0.28*	ND	Colony stimulating factor 3
FGFR3	2.54	=	Fibroblast growth factor receptor 3
MSX1	ND	0.67*	Msh homeobox 1
PDGFA	3.88***	=	Platelet-derived growth factor alpha
PHEX	0.38**	=	Phosphate-regulating neutral endopeptidase X-linked
RUNX2	=	1.95*	Runt-related transcription factor 2
SMAD1	0.69**	=	SMAD family member 1
SMAD4	1.50**	=	SMAD family member 4
SMAD7	1.54**	=	SMAD family member 7
SPARC	0.52***	1.50*	Secreted protein acidic, cystein rich / Osteonectin
TGFB1	1.86*	1.93*	Transforming growth factor beta 1
TGFB2	2.13**	6.12*	Transforming growth factor beta 2
TGFB3	0.47	0.53	Transforming growth factor beta 3
TUFT1	2.90**	=	Tuftelin 1
VEGFA	2.83***	=	Vascular endothelial growth factor A
VEGFC	0.36***	ND	Vascular endothelial growth factor C

Included are genes with: 2 > FC < 0.5 and if *P* < 0.05 also genes with 1.5 > FC < 0.66. Bold indicates genes with 2 > FC < 0.5 and *P* < 0.05. = indicates no difference in gene expression level. L-19; LNCaP-19, *ND* not detected

*P < 0.05, **P < 0.01, ***P < 0.001

**Fig. 4:**
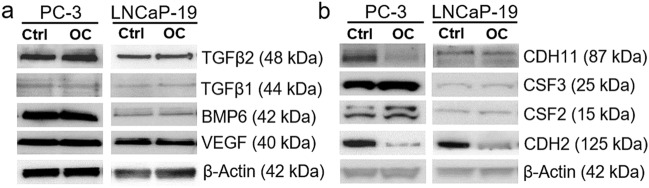
Protein analysis selected genes involved in osteogenesis **a** Growth factors regulating bone metabolism and tumor growth, and **b** proteins involved in osteoclast differentiation and cell adhesion in PC-3 and LNCaP-19 cells after co-culture with mature osteoclasts or control cells (unstimulated RAW 264.7 cells). β-actin was used as loading control

The increased expression of CSF2 in PC-3 cells after co-culture with osteoclasts, indicating an inhibiting effect on osteoclasts, was confirmed at the protein level, while CSF3 levels were unchanged. In addition, osteoclasts decreased both osteoblast cadherin (CDH11) and N-cadherin (CDH2) indicating a decreased capacity of attachment of the PC-3 cells to other cell types in the bone microenvironment. No altered expression of secreted factors promoting osteogenesis could be confirmed on the protein level (Fig. 4a, b).

## Discussion

Interaction with bone cells is important for prostate cancer cells to grow as metastases in the skeleton. The present study demonstrates direct influence of osteoclasts on prostate cancer cell proliferation and gene expression. In particular, genes regulating DNA repair, RNA modifications, and ER stress-induced apoptosis were affected. Further, osteoclasts influence osteolytic PC cells to a gene expression pattern characteristic for osteogenesis, thus stabilizing bone metabolism.

Osteoclasts have previously been attributed an important role in prostate cancer progression in bone, by degrading bone tissue and releasing tumor-promoting factors in the so-called vicious cycle. It is also known that prostate cancer cells directly promote differentiation of both osteoclasts and osteoblasts from their precursors [5, 40, 41]. However, a direct role of osteoclasts in regulating prostate cancer function has previously not been established. The present study suggests that mature osteoclasts, in comparison to macrophage precursor cells, increased proliferation and decreased apoptosis in prostate cancer cells. The effect was larger for osteoblastic prostate cancer cells compared to osteolytic and more dedifferentiated prostate cancer cells, in line with the effect of osteoblast stimuli in a previous study [25]. This indicates that these osteoblastic cells are more susceptible to bone cell stimulation regulating proliferation, compared to the osteolytic prostate cancer cells, which display overall more dedifferentiated features such as decreased AR expression. It may also indicate that osteoblastic prostate cancer cells are more sensitive to therapies aiming to inhibit proliferation, while cytotoxic strategies targeting stably proliferating cells may be more efficient in metastases dominated by osteolysis. Further, the phenotypical characteristics of bone remodeling has been linked to molecular phenotypes with impact on prognosis [42, 43].

In contrast to the proliferative response, osteolytic PC-3 cells displayed larger differences in overall gene expression after osteoclast stimulation compared to LNCaP-19. More genes were affected and consequently more GO terms describing their roles were identified. In general, few of classical tumor metastasis associated genes or pathways, such as those related to epithelial-mesenchymal transition, invasion or angiogenesis, were affected by co-culture with osteoclasts. Since the control cells in this setting are their macrophage precursor cells this might not be surprising. It is known that tumor infiltrating macrophages affect the progression of tumors and metastases [44], and supposedly their progeny, the osteoclasts, retain that ability, supporting a beneficial function of inhibiting the differentiation of or directly targeting this cell population in tumors. Despite the close relation between macrophages and osteoclasts, a large number of genes and functions were affected specifically by mature osteoclasts in the present study.

Of special interest in relation to cancer therapy are effects on DNA repair mechanisms. In both PC-3 and LNCaP-19, genes related to DNA-repair were overrepresented among affected genes. Several of the DNA repair genes upregulated by osteoclasts overlaps with the homologous recombination repair pathway (BRCA1, BRCA2, PALB2, BRIP1, and RAD51C), of which two (BRCA1 and PALB2) were shown to be affected also on the protein level in the present study. The decreased DNA damage, as demonstrated by lower γ-H2AX levels, in osteoclast-stimulated CRPC cells after exposure to UV-light supports that osteoclasts influence the response to DNA damage in CRPC cells. Deletion or mutation in DNA repair genes are suggested to indicate sensitivity to PARP-inhibitors, [45] as evaluated in the AMPLITUDE trial for prostate cancer (NCT04497844). However, the possible impact on sensitivity of different drugs needs to be investigated further, especially since a connection between sensitivity to PARP-inhibitors and AR signaling has been established [46].

Interaction between tumor cells and the bone microenvironment is necessary to convey the different phenotypes of the metastases defined by different prostate cancer cell types. Osteolytic prostate cancer cells promote osteoclast differentiation and activity while osteoblastic prostate cancer cells stimulate proliferation and differentiation of osteoblasts [5]. In addition, osteoblastic PC cells display osteomimetic properties such as expression of osteoblast specific markers and mineralization of bone matrix in vitro, features that were increased by stimulation by osteoblasts. In the present study, osteolytic PC cells were more sensitive than osteoblastic PC cells to osteoclast stimulation regarding gene expression related to bone metabolism. The parallel, i.e. that osteoblastic PC cells responded more to osteoblasts, was previously described, indicating a phenotype dependent gene regulation by bone cells [25]. However, only a few of the observed changes in the present study could be verified on the protein level, indicating that other regulatory mechanisms than RNA transcription, possibly provided by other parts of the bone microenvironment not present in the in vitro system, are important for the final phenotypic changes. Colony stimulating factor 2 (CSF2), which inhibits osteoclast differentiation [47], was still upregulated in PC-3 cells after osteoclast stimulation when assessed on the protein level, indicating a possible regulatory mechanism balancing the osteolytic progression. Together with the altered CSF2, the two cell adhesion proteins, osteoblast-cadherin (CDH11) and N-cadherin (CDH2) were downregulated, suggesting that osteoclasts decrease the tumor cell ability to adhere to mesenchymal cells in the microenvironment.

One pathway identified as significantly downregulated in PC-3 in this study was regulation by sterol regulatory element-binding protein 2 (SREBP2) including several enzymes involved in cholesterol synthesis. Cholesterol is a vital component in cell membranes and a precursor for steroid synthesis, including androgens. In prostate cancer, high cholesterol and cholesterol biosynthesis have been associated with for example progression [48], metastasis [49], and poor treatment response to taxanes [50]. Together with the downregulation of the cholesterol synthesis pathway by ostecoclasts in the present study, this would indicate a suppressive role of osteoclasts on aggressive features associated with cholesterol. However, a detectable effect on cholesterol levels needs to be confirmed in further studies.

Osteoclasts decreased gene expression related to ER stress induced apoptosis in both cell lines, identified both from the enriched GO terms and the list of the most affected genes. RNA expression for several genes, such as ATF3, ATF4, DDIT3, TRIB3, and CHAC1, involved in ER stress induced apoptosis was downregulated after co-culture with osteoclasts compared to the control cells, and both ATF3 and DDIT3 were confirmed as downregulated on the protein level. ER stress results in accumulation of un-folded proteins, which elicit either rescuing programs or activated apoptosis [51, 52]. The lower expression of genes associated with ER stress induced apoptosis may indicate that this mechanism for apoptosis induction was specifically decreased by osteoclasts. It may also suggest that osteoclasts decrease the sensitivity to drugs inducing cell death due to unfolded protein responses, highlighting an area for further studies regarding a possible protective role of osteoclasts for tumor cells in the bone microenvironment.

A limitation of the present study is the complexity of the co-culture system, where, in the osteoclast-stimulated situation, precursor cells are intermixed with the differentiated osteoclasts. To which extent the matured osteoclasts influence the remaining precursor cells, and how that affect their interaction with the tumor cells, as compared to the control situation, is not known. Thus, a contaminating effect by the remaining precursor cells in the osteoclast stimulation of tumor cells cannot be ruled out. Further studies are needed to identify the specific function of pure osteoclasts. Another limitation is the absence of other bone cells in the model system, thus the interpretations from the study must be further evaluated in the full complexity of the bone microenvironment. The present study does not identify the mechanism(s) by which osteoclasts act on tumor cells, an information that would be of great interest in terms of future targeting of this interaction. The putative upstream regulators suggested by the bioinformatic analysis may indicate certain targets, such as mTOR, for future studies in this regard.

In conclusion, the present study suggests that osteoclasts directly influence the growth and function of prostate cancer cells. In addition, osteoclasts affect gene expression pathways involved in DNA repair and apoptosis, indicating that they can influence the sensitivity to cytotoxic drugs. This broadens the putative role of therapeutic osteoclast inhibition to include combination therapies.

## Supplementary Information

Below is the link to the electronic supplementary material.

Supplementary file1 (PDF 855 KB)

## Data Availability

The datasets generated in and analyzed in the current study are available from the corresponding author on reasonable request.

## Abbreviations

CRPC: Castration-resistant prostate cancer
ADT: Androgen deprivation therapy
RANKL: Receptor activator of nuclear factor kappa-Β ligand
FDR: False discovery rate
ER: Endoplasmic reticulum
