# Supplementary material for: Osteoclasts directly influence castration-resistant prostate cancer cells
Source: Clin Exp Metastasis. 2022 Aug 16;39(5):801–14. doi: 10.1007/s10585-022-10179-2 (PMC9474581; doi:10.1007/s10585-022-10179-2)
Supplement: Supplementary file 1 — Supplementary file1 (PDF 855 KB) [file 10585_2022_10179_MOESM1_ESM.pdf]

SUPPLEMENTARY DATA

Supplementary figures

Figure S1

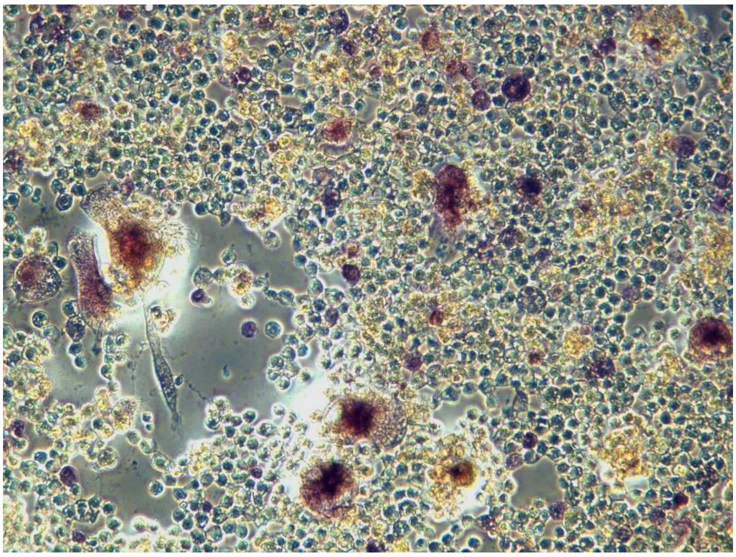

Supplementary figure S1. Staining with TRAP for detection of mature osteoclasts

Figure S2

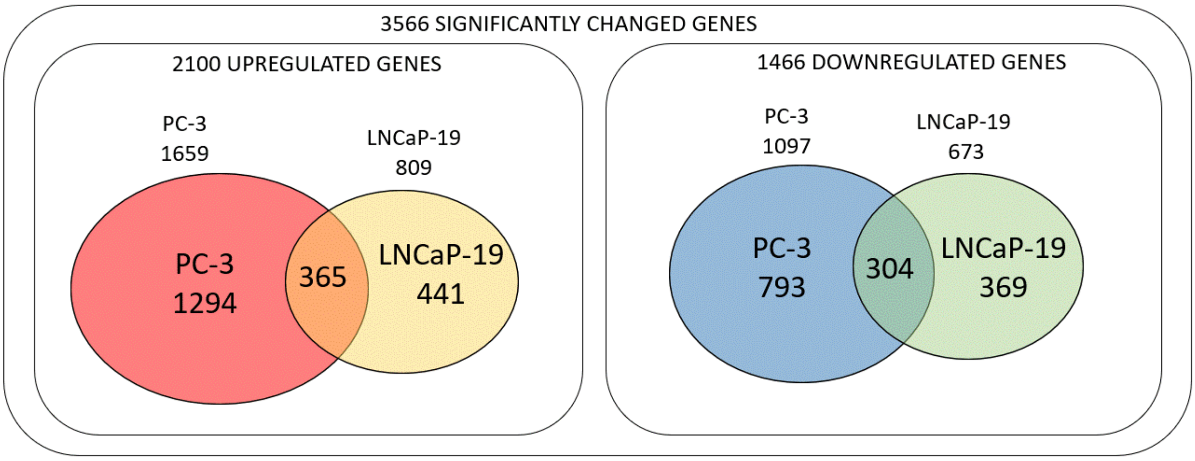

Supplementary figure S2. Overview of the significantly changed gene expression in PC-3 and LNCaP-19 after co-culture with mature osteoclasts compared to the untreated RAW264.7 control cells

Figure S3

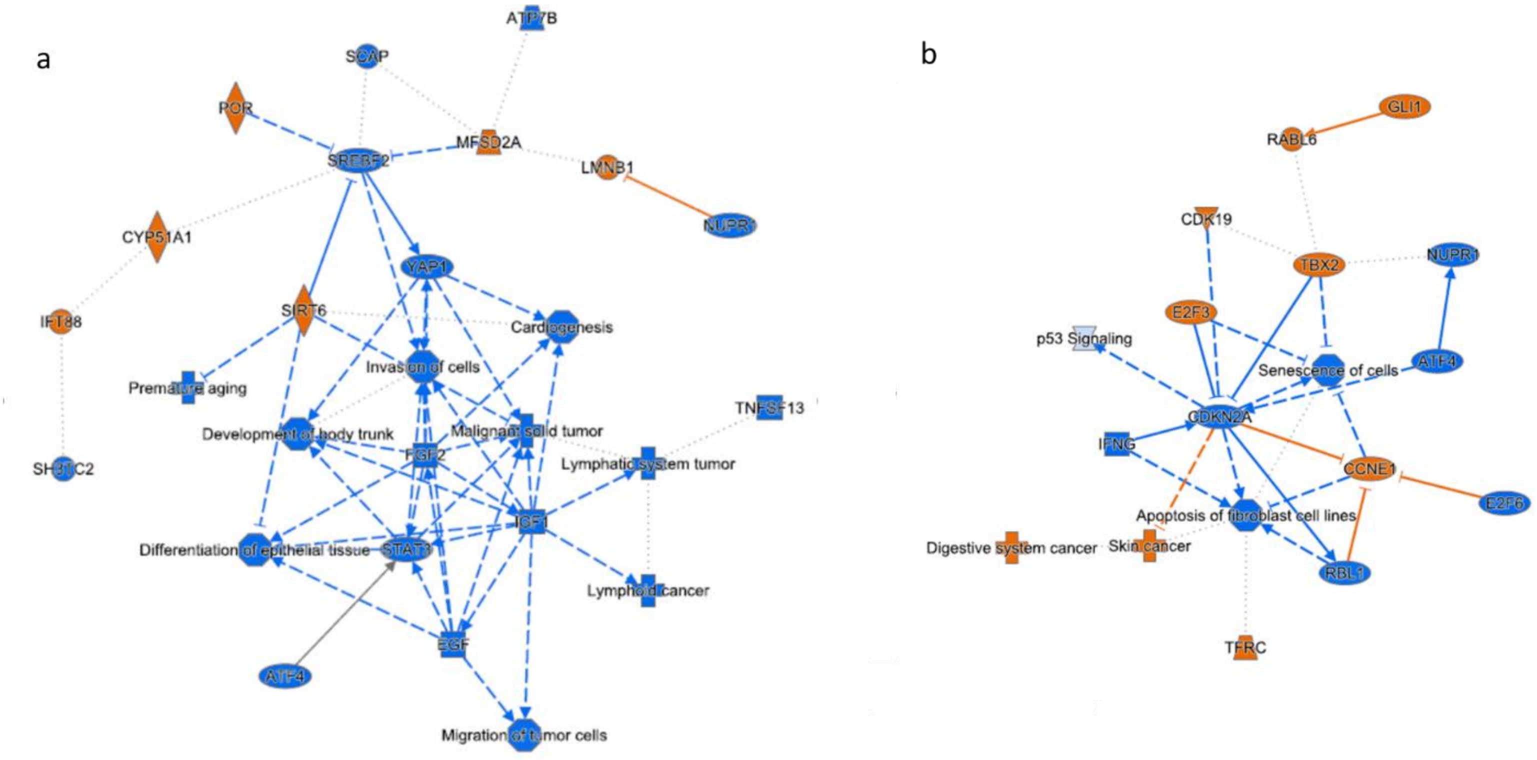

Supplementary figure S3. Graphical representation of significantly altered pathways identified by IPA Core Analysis of RNA expression in a) PC-3 and b) LNCaP-19 cell cultures co-cultured with mature osteoclasts compared to control.

IPA Legend for Figures S3, S4, S5 (for more details see https://qiagen.secure.force.com/KnowledgeBase/articles/Knowledge/Legend)

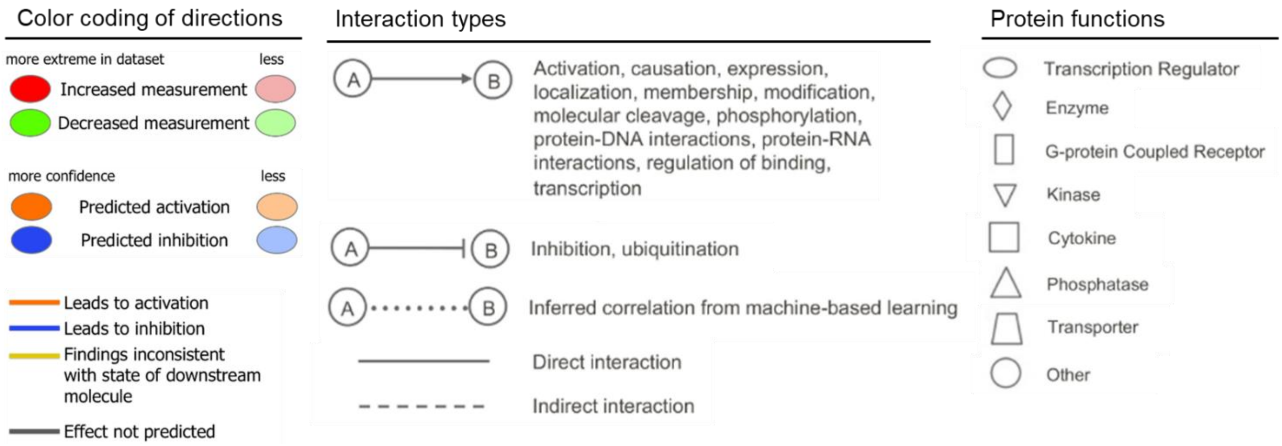

Figure S4

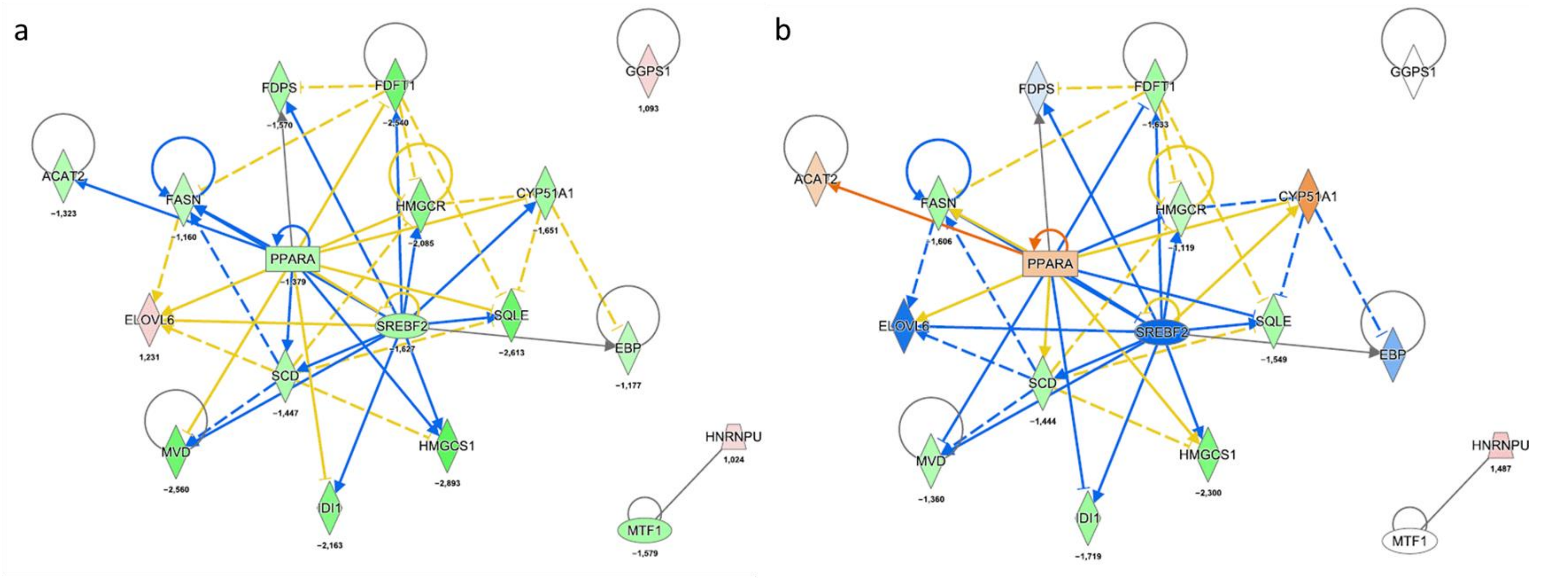

Supplementary figure S4. RNA expression changes in the SREB2F cholesterol pathway illustrated with IPA for a) PC-3 and b) LNCaP-19 cells co-cultured with osteoclasts. Numbers under genes show the fold change in expression in cells co-cultured with osteoclasts compared to control cells (unstimulated RAW 264.7).

Figure S5

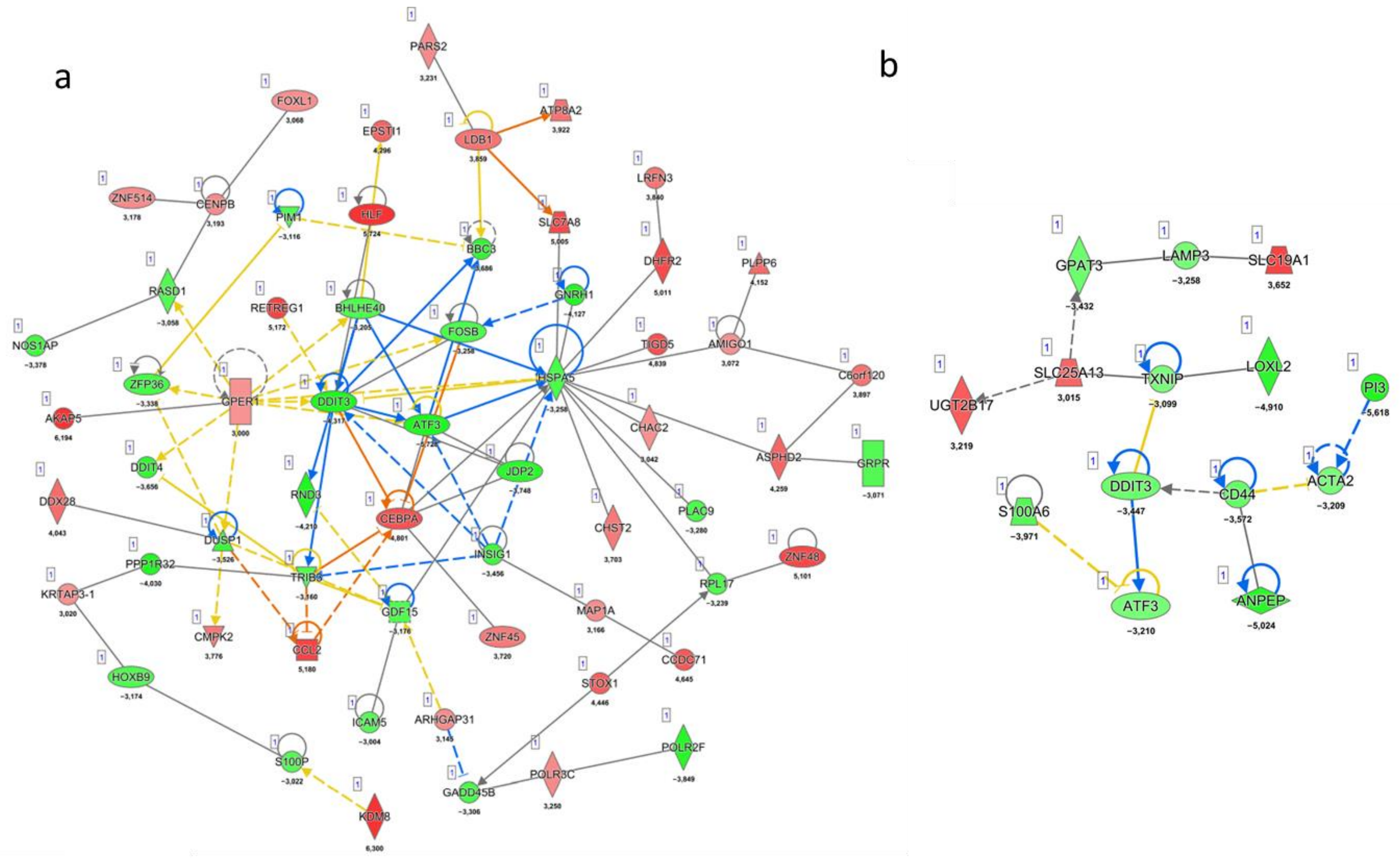

Supplementary figure S5. Illustration of clusters identified with QIAGEN IPA among the genes with largest expression changes (FC>3) in a) PC-3 and b) LNCaP-19 cells co-cultured with osteoclasts compared to untreated RAW 264.7 cells. Solid lines indicate a direct link is shown in publications in the Qiagen IPA database and dotted lines indicate an indirect link. Numbers under genes show the fold change in expression in cells co-cultured with osteoclasts compared to control cells.

Supplementary tables

Supplementary table 1. Antibodies for Western blotting

Target protein	Type	Company	Product number
ATF3	Rabbit polyclonal	Abcam	ab216569
BMP6	Rabbit monoclonal	Abcam	ab155963
BRCA1	Rabbit polyclonal	Abcam	ab238983
BRCA2	Rabbit polyclonal	Abcam	ab216972
DDIT3	Rabbit polyclonal	Proteintech Group	15204-1-AP
DDIT4	Rabbit monoclonal	Abcam	ab191871
FDFT1	Rabbit monoclonal	Abcam	ab195046
γ-H2AX [p Ser139]	Rabbit polyclonal	Novus Biologicals	NB100-384
G-CSF (CSF3)	Rabbit monoclonal	Abcam	ab181053
GM-CSF (CSF2)	Rabbit polyclonal	Abcam	ab9741
N-Cadherin (CDH2)	Rabbit monoclonal	Abcam	ab76011
OB-cadherin (CDH11)	Rabbit polyclonal	Abcam	ab151302
SP1	Rabbit monoclonal	Abcam	ab124804
SQLE	Rabbit polyclonal	Sigma-aldrich	HPA020762
SREBP2	Rabbit polyclonal	Abcam	ab30682
TGF beta 1	Rabbit monoclonal	Abcam	ab215715
TGF beta 2	Mouse polycloncal	Abcam	ab167655
VEGFA	Rabbit monoclonal	Abcam	ab214424
Loading controls			
β-Actin	Mouse monoclonal	Sigma-aldrich	A5316
β-Tubulin I	Rabbit monoclonal	Abcam	ab179511
GAPDH	Mouse monoclonal	Sigma-aldrich	GS9545

Supplementary table 2. Significant gene expression changes in cells co-cultured with osteoclasts compared to control cells within the cholesterol pathway.

Gene symbol	Entrez Gene Name	Entrez Gene ID for Human	PC3 Fold Change	LNCaP-19 Fold Change
ACAT2	acetyl-CoA acetyltransferase 2	39	-1,32	
CYP51A1	cytochrome P450 family 51 subfamily A member 1	1595	-1,65	
EBP	EBP cholestenol delta-isomerase	10682	-1,18	
ELOVL6	ELOVL fatty acid elongase 6	79071	1,23	
FASN	fatty acid synthase	2194	-1,16	-1,61
FDFT1	farnesyl-diphosphate farnesyltransferase 1	2222	-2,54	-1,63
FDPS	farnesyl diphosphate synthase	2224	-1,57	
GGPS1	geranylgeranyl diphosphate synthase 1	9453	1,09	
HMGCR	3-hydroxy-3-methylglutaryl-CoA reductase	3156	-2,08	-1,12
HMGCS1	3-hydroxy-3-methylglutaryl-CoA synthase 1	3157	-2,89	-2,3
HNRNPU	heterogeneous nuclear ribonucleoprotein U	3192	1,02	1,49
IDI1	isopentenyl-diphosphate delta isomerase 1	3422	-2,16	-1,72
MTF1	metal regulatory transcription factor 1	4520	-1,58	
MVD	mevalonate diphosphate decarboxylase	4597	-2,56	-1,36
PPARA	peroxisome proliferator activated receptor alpha	5465	-1,38	
SCD	stearoyl-CoA desaturase	6319	-1,45	-1,44
SQLE	squalene epoxidase	6713	-2,61	-1,55
SREBF2	sterol regulatory element binding transcription factor 2	6721	-1,63	

Supplementary table 3. The most significantly affected genes in prostate cancer cells by osteoclast stimulation based on false discovery rate

a. Most significantly upregulated genes in PC-3						b. Most significantly upregulated genes in LNCaP-19					
Over all top 20			Top 20 uniquely in PC-3*			Over all top 20			Top 20 uniquely in LNCaP-19*		
GeneID	log Fold-Chang	False Discovery Rate	GeneID	log Fold-Chang	False Discovery Rate	GeneID	log Fold-Chang	False Discovery Rate	GeneID	log Fold-Chang	False Discovery Rate
HOXB13 *U	3,46	2,91E-10	SHISA3	2,51	6,03E-07	HNRNPM	2,78	4,92E-09	DDX39A	1,60	3,84E-06
RBM15B *U	3,01	6,33E-09	MAGEF1	2,80	6,06E-07	SLC19A1	3,65	3,72E-08	TYMS	2,07	5,13E-06
INTS5 *U	3,29	4,85E-08	IMP3	2,74	6,80E-07	FUS	2,63	3,72E-08	APC2	4,00	7,16E-06
FAM111B	5,18	1,25E-07	NOP9	2,44	7,01E-07	SFPQ *U	2,26	3,72E-08	KEAP1	1,87	1,00E-05
TIGD5	4,84	1,26E-07	EXOC7	1,82	7,74E-07	NCOA5	3,49	4,32E-08	NRARP	1,91	1,25E-05
B3GALT6	3,07	1,55E-07	TMEM203	3,20	8,91E-07	SRSF3	2,07	1,25E-07	MCM7	1,66	1,38E-05
PRRT3 *U	5,38	1,67E-07	FEM1A	2,95	9,80E-07	HNRNPD *U	2,09	2,47E-07	FNDC10	2,20	1,38E-05
FOXRED2 *U	2,85	1,93E-07	ZBTB4	1,97	1,03E-06	LAD1	2,74	3,99E-07	CSE1L	1,36	1,39E-05
CTDSP1	2,45	2,64E-07	TMEM223	2,44	1,11E-06	SRSF2	2,29	4,55E-07	U2AF2	1,70	1,63E-05
**DBNL**	2,16	2,77E-07	ZNF48	5,10	1,11E-06	DHX9	2,57	6,95E-07	HNRNPAB	1,60	2,08E-05
TMEM127 *U	2,44	2,77E-07	SLC35C1	2,52	1,21E-06	SRSF1	2,89	1,06E-06	NME1	1,36	2,08E-05
POLR1A *U	2,76	2,77E-07	POGLUT1	3,35	1,34E-06	ZNF512B	2,85	1,06E-06	COMMD4	1,48	2,17E-05
MGAT2 *U	3,55	2,91E-07	IKBKE	2,99	1,44E-06	ZWINT	1,96	1,07E-06	SCGN	2,41	2,24E-05
FADD *U	2,94	2,98E-07	RRAGA	1,88	1,52E-06	MCM3	2,10	1,07E-06	WDR34	1,45	2,75E-05
PPP2R1B *U	2,49	3,09E-07	TCF19	2,52	1,59E-06	GINS2	2,10	1,07E-06	SFXN2	2,61	2,75E-05
FAM43A *U	2,45	3,21E-07	ZNF623	2,67	1,60E-06	UGT2B11 *	4,26	1,49E-06	EIF5A	1,64	2,78E-05
SLC12A2 *U	1,87	3,27E-07	ZNF518B	2,94	1,60E-06	ACPP *U	2,92	1,88E-06	BAG1	1,66	3,05E-05
CSTF2T *U	3,41	4,37E-07	RIOX1	3,26	1,84E-06	**DBNL**	1,79	1,88E-06	TSTA3	1,78	4,38E-05
C12orf43	3,01	5,01E-07	FZD8	3,99	1,88E-06	MCM2	1,89	2,53E-06	PPIF	1,40	4,48E-05
ACP2	3,58	6,03E-07	HOXA13	3,03	1,92E-06	PIGW	2,17	3,21E-06	HNRNPDL	1,34	5,37E-05

c. Most significantly downregulated genes in PC-3						d. Most significantly downregulated genes in LNCaP-19					
Over all top 20			Top 20 uniquely in PC-3*			Over all top 20			Top 20 uniquely in LNCaP-19*		
GeneID	log Fold-Chang	False Discovery Rate	GeneID	log Fold-Chang	False Discovery Rate	GeneID	log Fold-Chang	False Discovery Rate	GeneID	log Fold-Chang	False Discovery Rate
HSPA5 *U	-3,26	5,45E-11	DUSP1	-3,53	1,95E-08	**DDIT4**	-2,81	3,72E-08	SMIM14	-1,51	7,16E-06
**BBC3**	-3,69	7,26E-11	ANKRD10	-2,45	2,81E-08	**GDF15**	-2,89	3,72E-08	TRIM13	-1,58	8,73E-06
RND3	-4,21	1,66E-10	SNRK	-2,58	2,90E-08	**DDIT3**	-3,45	5,46E-08	KCNRG	-3,14	1,09E-05
**DDIT3**	-4,32	1,88E-10	PROSER2	-2,50	3,32E-08	ACTA2 *U	-3,21	8,34E-08	CBX4	-1,33	1,30E-05
**DDIT4**	-3,66	2,10E-10	NMT2	-2,44	3,77E-08	**GADD45B**	-2,97	1,08E-07	ESRP1	-1,86	1,35E-05
**ATF3**	-5,73	2,23E-10	PIM3	-2,45	6,40E-08	NDRG1	-2,46	1,08E-07	SNAPC3	-2,15	1,35E-05
**GADD45B**	-3,31	3,04E-10	LARP1B	-2,45	1,04E-07	TSC22D3	-2,71	1,46E-07	CDKN1B	-1,60	1,43E-05
**GDF15**	-3,18	3,76E-10	NEK2	-2,33	1,67E-07	**ATF3**	-3,21	1,25E-06	FAM102A	-1,58	1,53E-05
SESN2	-4,01	1,12E-09	KLF3	-2,95	2,63E-07	**SAT1**	-2,39	1,72E-06	TAOK3	-1,74	1,63E-05
TRIB3	-3,16	1,12E-09	MKNK2	-1,90	3,09E-07	KLHL24	-2,29	1,88E-06	TPM4	-1,56	1,63E-05
**INSIG1**	-3,46	2,84E-09	FOXK1	-1,97	3,13E-07	STC2	-2,36	1,88E-06	SVIL	-2,70	2,08E-05
GRB7	-2,91	5,09E-09	KLF7	-2,87	3,24E-07	SLC9A7 *U	-1,71	1,98E-06	FKBP14	-2,42	2,46E-05
**SAT1**	-2,88	5,09E-09	IRX2	-2,22	3,39E-07	MYLK *U	-2,30	2,40E-06	MVP	-3,37	3,05E-05
SLC5A3 *U	-2,81	5,94E-09	NEURL1B	-2,81	3,48E-07	CHAC1	-2,97	3,21E-06	NEDD9	-1,73	3,30E-05
DUSP5 *U	-2,74	7,99E-09	AKAP12	-1,85	3,55E-07	JMY	-2,34	3,85E-06	WSB1	-1,84	3,52E-05
FDFT1	-2,54	1,01E-08	CCN1	-2,70	3,55E-07	**BBC3**	-1,84	4,34E-06	LPIN1	-1,58	4,17E-05
BRD2 *U	-2,21	1,20E-08	PRKCZ	-1,80	3,55E-07	**INSIG1**	-2,21	4,34E-06	PPP1R3B	-1,96	4,53E-05
GABARAPL1	-2,72	1,28E-08	UGCG	-2,91	4,13E-07	SETD5	-1,67	5,33E-06	TRIM4	-1,60	5,37E-05
SLC4A7	-2,50	1,52E-08	ING1	-1,87	4,26E-07	TXNIP	-3,10	5,58E-06	PPM1K	-1,57	7,39E-05
ATF4	-2,03	1,95E-08	H2BC21	-2,64	4,42E-07	YPEL5	-1,79	6,83E-06	TNFRSF1A	-1,46	8,06E-05

* in addition to those qualifying into the over all top 20 list

*U: uniquely up/downregulated in PC-3 or LNCaP-19, respectively

Bold: Qualified into overall top20 in both cell lines
